# Highly Branched Poly(5-amino-1-pentanol-*co*-1,4-butanediol diacrylate) for High Performance Gene Transfection

**DOI:** 10.3390/polym9050161

**Published:** 2017-05-01

**Authors:** Ming Zeng, Dezhong Zhou, Singwei Ng, Jonathan O’Keeffe Ahern, Fatma Alshehri, Yongsheng Gao, Luca Pierucci, Udo Greiser, Wenxin Wang

**Affiliations:** 1 Charles Institute of Dermatology, University College Dublin, Dublin 4, Ireland; ming.zeng@ucdconnect.ie (M.Z.); jonathan.o-keeffe-ahern@ucdconnect.ie (J.O.A.); fatma.alshehri@ucdconnect.ie (F.A.); yongsheng.gao@ucdconnect.ie (Y.G.); luca.pierucci@gmail.com (L.P.); dr.udo.greiser@gmail.com (U.G.); 2 Department of Dermatology, the First Affiliated Hospital of Anhui Medical University, Hefei 230022, China; 3 School of Food Science and Environmental Health, College of Sciences and Health, Dublin Institute of Technology, Dublin 2, Ireland; d13122539@mydit.ie

**Keywords:** non-viral vectors, gene therapy, transfection efficiency, cytotoxicity, nanoparticles, poly(β-amino ester)s

## Abstract

The top-performing linear poly(β-amino ester) (LPAE), poly(5-amino-1-pentanol-*co*-1,4-butanediol diacrylate) (C32), has demonstrated gene transfection efficiency comparable to viral-mediated gene delivery. Herein, we report the synthesis of a series of highly branched poly(5-amino-1-pentanol-*co*-1,4-butanediol diacrylate) (HC32) and explore how the branching structure influences the performance of C32 in gene transfection. HC32 were synthesized by an “A2 + B3 + C2” Michal addition strategy. Gaussia luciferase (Gluciferase) and green fluorescent protein (GFP) coding plasmid DNA were used as reporter genes and the gene transfection efficiency was evaluated in human cervical cancer cell line (HeLa) and human recessive dystrophic epidermolysis bullosa keratinocyte (RDEBK) cells. We found that the optimal branching structure led to a much higher gene transfection efficiency in comparison to its linear counterpart and commercial reagents, while preserving high cell viability in both cell types. The branching strategy affected DNA binding, proton buffering capacity and degradation of polymers as well as size, zeta potential, stability, and DNA release rate of polyplexes significantly. Polymer degradation and DNA release rate played pivotal parts in achieving the high gene transfection efficiency of HC32-103 polymers, providing new insights for the development of poly(β-amino ester)s-based gene delivery vectors.

## 1. Introduction

Gene therapy is broadly defined as the procedure used to genetically modify the target cells with the intention of altering gene expression to prevent, relieve, or reverse pathological genetic deficiency conditions including both inherited and acquired diseases. Over the past two decades, the high efficacies of trans-gene expression by viral vectors has made them attractive and advanced their applications in numerous clinical trials. However, vector-host safety profiles are adversely affected by side effects of viral vectors, including immunogenicity [1], carcinogenesis [2] and broad tropism [3]. The technology of viral gene therapy is also hampered by its limited nucleic acid packaging ability [4] and difficulties associated with the scalability of virus production [5]. In the area of non-viral gene delivery systems, the utilization of cationic polymers in clinical applications has increased from 2004 to 2017 while viral products saw a dramatic decrease [6]. The efforts to develop enhanced polymeric vectors resulted in major advances such as larger gene delivery capacities, lower immunogenicity and easier manufacturing processes compared to the current viral vector technology [7,8]. Encouragingly, linear poly(β-amino ester)s (LPAEs), one type of the most versatile polymeric vectors first developed in 2000 [9], demonstrated its superiority in gene transfection in comparison with other non-viral vectors. After establishing a polymer combinational library, over 2500 LPAEs have been synthesized and screened [10,11]. The backbone, side chain, terminal group and degradable linkages in poly(β-amino ester)s (PAEs) can be adjusted easily [12,13,14], the polymer/DNA nanoparticles are biodegradable with a half-life between 1 and 7 h in an aqueous environment [15], and they are not cytotoxic. These polymeric nanoparticles can be fabricated, lyophilized, frozen and stored for at least two years without a reduction of efficiency [16], suggesting that PAEs may provide promising alternatives for future translational applications. Among all the LPAEs, the top-performing poly(5-amino-1-pentanol-*co*-1,4-butanediol diacrylate) (C32) series have been shown to achieve comparable efficacy to adenovirus in human primary cells [17] and umbilical vein endothelial cells [18]. Notably, among the end-modified C32 polymeric vectors, the one end-capped with 1,3-diaminopropane (termed as LC32-103), has been identified to be an extraordinarily efficient vector both in vitro and in vivo in a wide range of biomedical applications [17,19,20]. 

Structure is a crucial parameter determining aspects of a gene vector such as efficiency and safety. Branched polymers have many attractive properties over linear polymers including a three-dimensional (3D) structure with multiple functional terminal groups and relatively high molecular flexibility [21,22], offering many reaction units for further conjugation. To date, branched polyethyleneimine (PEI) [23], branched poly-l-lysine (PLL) [24,25], branched poly(2-dimethylaminoethyl methacrylate) (PDMAEMA) [26] and branched glycopolymers [27] have been proven to be superior to their linear rivals in transfection efficiency. Although the linear structure of linear C32 (LC32) is limited by only two end groups, branched C32 (HC32) has rarely been synthesized. There are challenges in the synthesis of highly branched poly(β-amino ester)s (HPAEs) due to their very restrictive reaction conditions (e.g., high pressure and presence of a special catalyst [28], or unequal reactivity of the multiple functional groups in monomers [29]). Recently, by introducing trimethylolpropane triacrylate (TMPTA), a branching monomer, HPAEs were synthesized from the same library of LPAE monomers by our group. HPAE-mediated transfection efficiency was enhanced up to 8521-fold over the corresponding linear counterparts and the commercially available reagents PEI and SuperFect [30].

To our knowledge, there is no study on HC32 with high degree of branching. Therefore, branching of the LC32 needs to be further explored to investigate how the structure affects the transfection efficiency. Herein, based on LC32-103, we successfully developed a series of HC32-103 polymers with diverse degrees of branched structures. Furthermore, physicochemical properties, size, zeta potential of polyplexes, proton buffering capacity, DNA binding affinity, and polyplex stability in the presence of serum and salts were investigated to understand the underlying mechanism behind HC32 in gene delivery. Finally, utilizing Gaussia luciferase (Gluciferase) and green fluorescent protein (GFP) coding plasmid DNA as reporter genes, the performance of gene delivery of HC32 was further compared with LC32-103, placing emphasis on both transfection efficiency and cytotoxicity.

## 2. Materials and Methods 

### 2.1. Materials

Chemicals 1,4-butanediol diacrylate (C, VWR, Dublin, Ireland, 98%), 5-amino-1-pentanol (32, Sigma, Dublin, 99%), 1,3-diaminopropane (103, Sigma, Dublin, Ireland, 99%), trimethylolpropane triacrylate (Sigma, Dublin, 99%), lithium bromide (LiBr, Sigma, Dublin, Ireland, 99%), solvents dimethyl sulfoxide (DMSO, Sigma, Dublin, Ireland, 99%), dimethylformamide (DMF, Fisher Scientific, Dublin, Ireland, 99%), diethyl ether (Sigma, Dublin, Ireland, 99%), deuterated chloroform (CDCl_3_, Sigma, Dublin, Ireland, 99.9%), Hank’s balanced salt solution (HBSS, Sigma, Dublin, Ireland), branched polyethyleneimine (PEI, *M*_w_ = 25 kDa, Sigma, Dublin, Ireland), SuperFect (Qiagen, Dublin, Ireland), BioLux^TM^ Gaussia Luciferase Assay Kit (New England Biolabs, Dublin, Ireland) and Alamarblue Assay Kit (Invitrogen, Dublin, Ireland) were used as received. Sodium acetate (Sigma, Dublin, Ireland, pH 5.2 ± 0.1, 3 M) was diluted to 0.025 M prior to use. Picogreen was purchased from Life Technologies (Dublin, Ireland). Cell culture Dulbecco’s modified Eagle Medium (DMEM) was purchased from Sigma (Dublin, Ireland). Keratinocyte Growth Medium 2 (c-20011) was purchased from PromoCell (Dublin, Ireland). Penicillin-steptomycin was purchased from Thermo Fisher Scientific (Dublin, Ireland). Fetal bovine serum (FBS, Gibco, (Dublin, Ireland) was filtered through 0.2 μm filters before use. Cell-secreted Gaussia princeps luciferase plasmid (pCMV-GLuc) and GFP plasmid (pCMV-GFP) were obtained from New England Biolabs, London, UK.

### 2.2. Polymer Synthesis 

LC32 and HC32 base polymers (polymers prior to end-capping) were synthesized first. Monomer feed ratios for the synthesis of LC32 and HC32 base polymers are listed in Table S1. Taking HC32-10%-103 synthesis as an example, typically, 1,4-butanediol diacrylate (2 mmol, 0.396 g), trimethylolpropane triacrylate (0.2 mmol, 0.0592 g) and 5-amino-1-pentanol (2 mmol, 0.206 g) are dissolved in 1 mL DMSO, and the reaction occurs at 90 °C. Agilent 1260 Infinite gel permeation chromatography (GPC) was used to monitor the evolution of molecular weight (*M*_w_). The reaction was stopped by diluting the mixture to 100 mg/mL with DMSO when *M*_w_ was approaching 10,000 Da. 288 μL (2.64 mmol) 1,3-diaminopropane was added to end-cap the acrylate terminated base polymer at room temperature for 48 h. After that, polymers were precipitated into diethyl ether three times and dried under vacuum for 48 h before being stored at −20 °C. 

### 2.3. Molecular Weight Measurements 

Molecular weight (*M*_w_), polydispersity index (PDI) and Mark-Houwink alpha parameter (MH Alpha) of polymers were determined by GPC equipped with a refractive index detector (RI), a viscometer detector (VS DP) and a dual angle light scattering detector (LS 15° and LS 90°). To monitor the *M*_w_ of polymers during the polymerization process, 20 μL of the reaction mixture was collected at different time points, diluted with 1 mL of DMF, filtered through a 0.2 μm filter and then measured by GPC. The columns (PolarGel-M, Edinburgh, UK, 7.5 mm × 300 mm, two in series) were eluted with DMF and 0.1% LiBr at a flow rate of 1 mL/min at 60 °C. Columns were calibrated with linear poly(methyl methacrylate) (PMMA) standards. 

### 2.4. Proton Nuclear Magnetic Resonance (^1^H NMR)

Chemical structure and composition of polymers were confirmed with ^1^H NMR. Polymer samples were dissolved in CDCl_3_. Measurement were carried out on a Varian Inova 400 MHz spectrometer (Edinburgh, UK) and reported in parts per million (ppm) relative to the response of the solvent (7.24 ppm) or to tetramethylsilane (0.00 ppm).

### 2.5. Acid-Base Titration

To determine the proton buffering capacity, acid-base titration was conducted. 10 mg polymers were dissolved in DMSO to 100 mg/mL stock solution and then diluted with deionized water to 0.2 mg/mL, the pH value was initially adjusted to 10.0 using 1 M NaOH and then titrated to 3.0 with HCl (0.01 M). The pH values were determined with a Sartorius PB-10 pH meter and the increment was 100 μL. 

### 2.6. Picogreen Assays

DNA binding affinity of polymers was measured with Picogreen assays. Briefly, polymers were dissolved in DMSO to 100 mg/mL. According to the *w*/*w* ratio, DNA and polymers were diluted with 30 μL of sodium acetate buffer, mixed by vortex for 15 s, and allowed to incubate for another 10 min. Two micrograms of DNA was used for each sample preparation. Then, 60 μL of Picogreen solution, which was prepared according to supplier’s instructions, was added and allowed to incubate for another 5 min. To a 96-well plate, 200 μL of DMEM (without serum) was added, and then 30 μL of the polyplex solution was added. Fluorescence measurements were carried out with a plate reader with an excitation at 490 nm and an emission at 535 nm.

### 2.7. Size and Zeta Potential of Polyplexes

Typically, 1 μg of DNA was used for each sample. According to the polymer/DNA weight ratio (*w*/*w*), DNA and the required polymer were dissolved in 40 μL of sodium acetate, respectively. Then, the polymer solution was added to the DNA solution and mixed by vortex for 15 s. The mixture was kept still for 10 min to formulate polyplexes. After that, 1 mL of deionized water was added and polyplex sizes and zeta potentials were measured with a Malvern Instruments Zetasizer (Nano-2590) (Malvern, UK) at a 90° scattering detector angle. All the measurements were repeated four times.

### 2.8. Polyplex Stability Measurements

To measure polyplex stability in the presence of serum, polyplexes were prepared as mentioned above and then FBS was added to the polyplex solution; the final FBS concentration was 5% or 10%. Polyplex sizes were measured immediately, as well as 30, 60 and 120 min post-incubation. To measure polyplex stability under high ion concentration, sodium chloride (NaCl) solution was added into the polyplex solution, and the final concentration was 300 or 600 mmol, respectively. Polyplex sizes were measured at 0, 30, 60 and 120 min post-incubation. All the measurements were repeated four times.

### 2.9. Polymer Degradation

Polymers were first dissolved in DMSO to 100 mg/mL and then diluted with deionized water to 1 mg/mL. The mixtures were incubated at 37 °C under stirring. At 0, 1, 2 and 4 h post-incubation, 10 mL of the polymer solution was taken out and freeze-dried immediately, *M*_w_ of the degraded products were measured as mentioned above. The percentage of degradation was defined as the molecular weight of the degraded polymers divided by the *M*_w_ of the original polymers. 

### 2.10. DNA Release

To measure the DNA release at different time intervals, polyplexes were prepared as mentioned above in sodium acetate buffer and then diluted with deionized water. Fluorescence was measured with Picogreen assays as mentioned immediately, 1, 2 and 4 h post-incubation. 

### 2.11. Cell Culture

The human cervical cancer cell line HeLa was cultured in DMEM containing 10% FBS and 1% Penicillin/Streptomycin (P/S). RDEBK cells were cultured in keratinocyte growth medium 2 (c-20011 pROMOCELL) with 1% Penicillin-steptomycin. Cells were cultured at 37 °C, in a humid incubator with 5% CO_2_, under standard cell culture techniques.

### 2.12. Transfection Experiments

Prior to transfection, cells were seeded on 96-well plates at a density of 1 × 10^4^ cells/well in 100 μL media and cultured until 70–90% confluence. We optimized the commercial transfection reagents PEI and SuperFect according to the manufacturer’s instructions, and also referred to one previous publication [26]. The commercial reagents were used in optimized formulations: *w*/*w* = 3:1 (PEI) or *w*/*w* = 9:1 (SuperFect). A measure of 0.5 μg DNA was used for both cell types. Polyplexes were prepared as mentioned below: Briefly, the polymer and DNA solutions were diluted with sodium acetate buffer to equal volumes (10 μL) according to the *w*/*w* ratios. Then, polymer solutions were added to the DNA solution, vortexed for 10 s and allowed to stand for 10 min. Cell culture media was then added to increase the volume of the polyplex solution to 100 μL. The media in the wells of the cell culture plates was removed quickly and the polyplex solution was added. Four hours later, the transfection medium was replaced with fresh medium and the cells were cultured for another 44 h.

### 2.13. Evaluation of Gene Transfection Efficiency Using Gluciferase Assays and GFP Expression

At 48 h post-transfection, using Gluciferase DNA, measurements of the Gluciferase activity were carried out as per the provided protocol using a SpectraMax M3 plate reader (Dublin, Ireland) with Gluciferase activity directly detected in the cell supernatant and plotted in terms of relative light units (RLU). Measurements were performed in quadruplicates with error bars indicating ± standard deviation. The GFP expression was observed and imaged by an inverted fluorescence microscope (Olympus IX81, Dublin, Ireland). 

### 2.14. Cytotoxicity Assessment after Transfection Using Alamarblue Assay

To perform Alamarblue assay, cell supernatants were first removed and then cells were washed with HBSS, followed by the addition of 10% Alamarblue reagent in HBSS. Living, proliferating cells maintain a reducing environment within the cytosol of the cell, converting the non-fluorescent ingredient resazurin in Alamarblue to the highly fluorescent compound resorufin. This reduction results in a color change from blue to light red and allows for the quantitative measurement of cell viability based on the increase in overall fluorescence and color of the media. The Alamarblue solution from each well was transferred to a fresh flat-bottomed 96-well plate for fluorescence measurements at 590 nm. Control cells without any treatment were used to normalize the fluorescence values and plotted as 100% viable. Measurements were performed in quadruplicates with error bars indicating ± standard deviation.

### 2.15. Statistics

All quantitative data were analyzed using GraphPad Prism (v.5, GraphPad Sofeware, San Diego, CA, USA). D’Agostino and Pearson omnibus normality tests were used to determine normal distribution. A one-way ANOVA was used where normal distribution was evident, followed by Tukey’s post hoc test. *p* values < 0.05 was considered to be statistically significant. All quantitative data were expressed as mean ± standard deviation.

## 3. Results and Discussion

### 3.1. Polymers Synthesis and Characterization

**Scheme 1:**
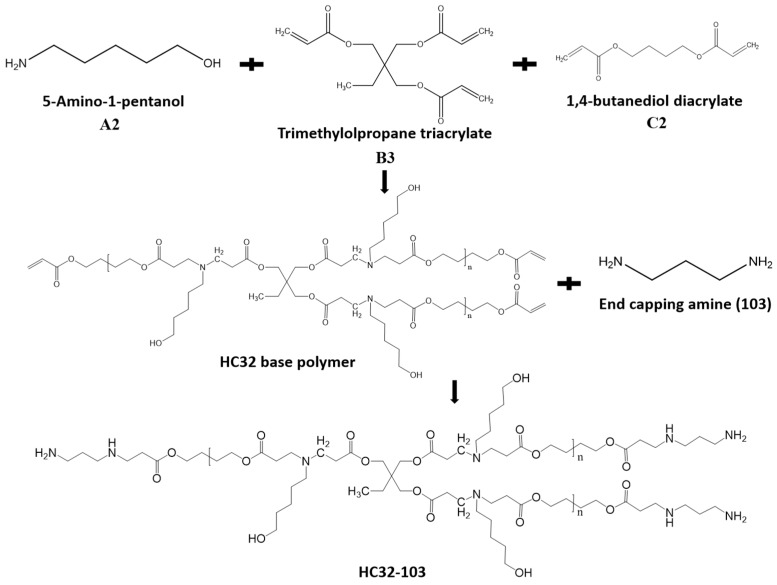
The synthesis of HC32-103 via an “A2 + B3 + C2” Michael addition approach. Acylate-terminated HC32 base polymers were first synthesized by copolymerization of A2 type monomer (5-Amino-1-pentanol), B3 type monomer (trimethylolpropane triacrylate) and C2 type monomer (1,4-butanediol diacrylate). In the second step, base polymers were further terminated with 1,3-diaminopropane (103) to synthesize HC32-103.

To validate our hypothesis, linear C32-103 were first synthesized by copolymerization of 5-amino-1-pentanol and 1,4-butanediol diacrylate without any branching monomers, and further end-capped with 1,3-diaminopropane. HC32 base polymers were synthesized via an “A2 + B3 + C2” Michael addition approach, which allows the branching triacrylate monomers to combine the linear segments, and then end-capped with 1,3-diaminopropane (Scheme 1). The monomer feed ratios for the synthesis of LC32 and HC32 polymers are summarized in Table S1. The stoichiometric ratio of the acrylates to amine was kept at 1.15:1.0 while varying the feed ratio of trimethylolpropane triacrylate to 1,4-butanediol diacrylate (0.1:1, 0.24:1, 0.43:1, 0.73:1, mole ratio). Consequently, a variety of branching HC32 polymers were prepared with a branching degree code of 10%, 24%, 43% and 73%, respectively. All HC32 were synthesized in a controlled manner without gelation or crosslinking, and can be dissolved very well in a variety of organic solvents and acid solutions at high concentration. 

The *M*_w_, PDI and MH plot alpha values of polymers are summarized in Table S2, and the GPC curves are shown in Figure S1a. The results show that all the branched polymers have higher Mw and PDI than LC32. As the branching degree increases, the MH plot alpha value of HC32 polymers decreases, indicating more branched structures. ^1^NMR spectra further confirm the success of the copolymerization of triacrylate and diacrylate with amine (Figure S1b–f). Meanwhile, the absence of the characteristic vinyl group signal peaks indicates the success of end-capping of based polymers with diamine by the Michael addition. All these results demonstrate that the “A2 + B3 + C2” Michael addition can be used to synthesize HPAEs with high branching degrees in a controlled manner, and that the branching degree can be adjusted easily by simply varying the feed ratio of triacrylates to diacrylates.

### 3.2. Biophysical Properties of LC32/DNA and HC32/DNA Polyplexes

**Figure 1:**
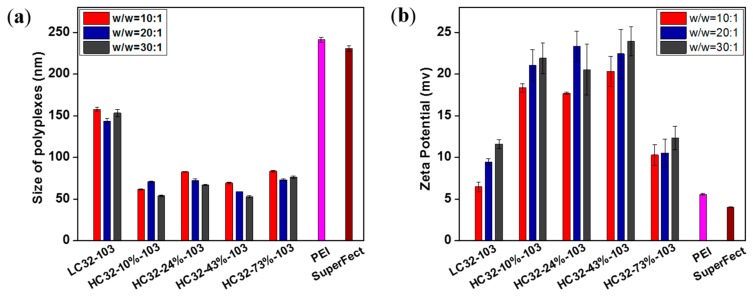
Size and potential of polyplexes formulated with different *w*/*w* ratios measured by Dynamic Light Scattering (DLS): (**a**) Size of polymer/DNA polyplexes; (**b**) Zeta potential of polyplexes.

The DNA condensation ability of LC32 and HC32 was tested using Picogreen assays. As shown in Figure S2, at the polymer/DNA weight ratio (*w*/*w*) of 20:1, all the polymers show high relative DNA binding affinity. This is because the diamine 103 was used to end-cap the base polymers, and the residual terminal primary amine can impart on the polymers a high positive charge. In general, the HC32-103 polymer series show higher DNA binding affinity than the LC32-103 counterpart, which is possibly due to the branching structure which imparts HC32-103 polymers with more terminal primary amine groups. Interestingly, the HC32 with high branching degrees (43% and 73%) does not show obvious differences in relative DNA binding affinity. The strong DNA binding affinity leads the HC32-103 polymers to condense DNA to formulate small polyplexes with positive zeta potentials. It is well known that the size and zeta potential of polyplexes exert a critical role on the cellular uptake process. Size and zeta potential of all polymer/DNA polyplexes at the three *w*/*w* ratios (10:1, 20:1 and 30:1) were measured using dynamic light scattering (DLS). As shown in Figure 1 and Table S3, all the polyplexes have small, uniform but obviously different sizes, with a PDI between 0.08 and 0.43, while LC32-103, PEI and SuperFect polyplexes exhibited sizes of around 150, 240 and 230 nm—much larger than that of the HC32-103/DNA polyplexes. The branching structure conferred HC32-103/DNA polyplexes with less than 90 nm of diameter, leading to around a 1/3 and 2/3 decrease in size, in comparison of that of the linear polymer LC32-103, PEI and SuperFect. Similarly, polyplexes in the HC32-103 group also illustrated higher zeta potential than the LC32-103 and commercial reagent groups. Interestingly, among the HC32-103 polymers, HC32-73%-103 with the highest branching degree led to a significant lower zeta potential. Compared with the HC32-103 group, the lowest potential values observed in LC32/DNA, PEI/DNA and SuperFect/DNA polyplexes were 6.5 (*w*/*w* = 10:1), 5.6 and 4.1 mv, respectively. The stability of the various polyplexes in the presence of serum was further tested. As shown in Figure S3, all the HC32/DNA polyplexes exhibit much higher stability than the LC32/DNA counterparts. In the presence of 5% or 10% serum, sizes of the LC32/DNA polyplexes increased 3–4 times and was up to 640 nm after 2 h incubation. In contrast, although increased, the sizes of the HC32-24%-103/DNA, HC32-43%-103/DNA, HC32-73%-103/DNA still remained lower than 300 nm. A similar trend of polyplex sizes was also observed in the presence of NaCl (Figure S4). These results demonstrate that the branching can significantly improve polyplex stability and in general, the higher branching degree leads to higher polyplex stability in serum or salt conditions. 

### 3.3. Transfection Efficiency and Cytotoxicity Measurement

**Figure 2:**
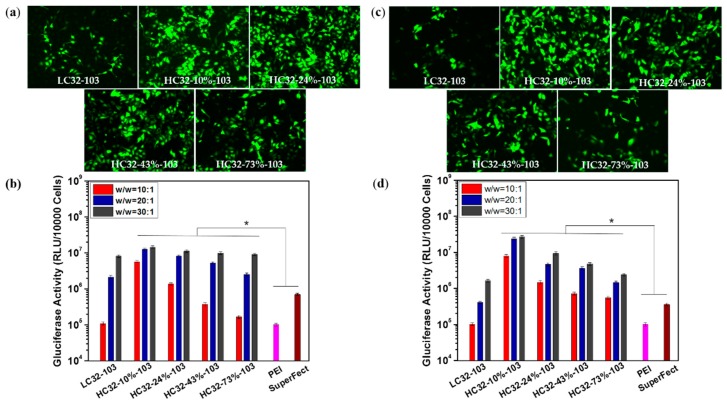
Transfection efficiency of LC32/DNA, HC32-103/DNA, PEI and SuperFect/DNA polyplexes formulated with different *w*/*w* ratios 48 h post-treatment: (**a**) GFP expression of HeLa cells treated with polyplexes; (**b**) Gluciferase activity of HeLa cells after treatment with polyplexes; (**c**) GFP expression of RDEBK cells; (**d**) Gluciferase activity of RDEBK cells after treatment with polyplexes, 40× magnification. Data points marked with an asterisk are statistically significant relative to the PEI and SuperFect group (*p* < 0.05).

To evaluate the transfection efficiency and biological compatibility of LC32-103 and HC32-103 polymers, transfections were performed using human cervical cancer cells (HeLa) and human skin cells (RDEBK). Polyplexes were formulated with polymer/DNA at *w*/*w* ratios of 10:1, 20:1 to 30:1. Two commercial dendritic gene transfection reagents, branched PEI (*M*_w_ = 25,000) and SuperFect, were used as positive controls according to optimized protocols. As illustrated in Figure 2**,** overall, the HC32-103 polymer series showed higher Gluciferase activity than the LC32-103 counterpart at all three tested *w*/*w* ratios, demonstrating up to 51.6-fold higher Gluciferase activity level in Hela cells and 77.3-fold higher in RDEBK cells compared with the linear counterpart LC32-103 (Figure 2). In addition, the HC32-103/DNA polyplexes mediated 1 to 2 orders of magnitude higher transfection efficacy than PEI and SuperFect/DNA polyplexes, up to 141.2-fold and 20.7-fold higher Gluciferase activity levels than PEI/DNA and SuperFect/DNA polyplexes in Hela cells and 265.4-fold and 75.2-fold higher than PEI/DNA and SuperFect/DNA polyplexes in RDEBK cells. The optimal formulation was observed in HC32-10%-103 at the *w*/*w* of 30:1, exhibiting the highest gene transfection efficiency. The gene transfection performance of all polymers and commercial reagents was further confirmed by the GFP expression. Similarly, in comparison with other counterparts, HC32-10%-103 exhibited a much higher fluorescence intensity than the LC32-103 polymer. Interestingly, as the branching degree of HPAEs increases, transfection efficiency showed a downward trend. 

The abovementioned Picogreen assays revealed that all the branched polymers have similar DNA binding ability (Figure S2), DLS results also showed that with the increase of branching degree, HC32-103 polymers exhibited similar sizes and positive zeta potentials (Figure 1). To decipher why the higher branching degree leads to lower gene transfection, we further conducted acid-base titration, given that the proton buffering capacity of polymers significantly affects their endosomal escape ability and ultimately gene transfection. As shown in Figure S5, basically all the HC32-103 polymers show similar proton buffering capacity between pH 5.2 and 7.4, which demonstrates that the difference in gene transfection efficiency of the various HC32-103 polymers was not derived from the proton buffering capacity. We further determined the polymer degradation profile with GPC (Figure S6). As expected, the polymer degradation rate decreases with the branching degree. After 4 h incubation, molecular weight of the HC32-10%-103 was only 12% of its original molecular weight, in contrast to 22%, 34% and 48% with the HC32-24%-103, HC32-43%-103 and HC32-73%-103, respectively. Accordingly, the DNA release profile measured by Picogreen showed that HC32-10%-103 has the fastest release rate (Figure S7). Previous studies have revealed that the vector degradation rate would significantly affect gene transfection efficiency; a too slow vector degradation would lead to low DNA release efficiency while a too fast vector degradation would result in amateur DNA release [7,15]. We speculate that here the 10% branching degree leads HC32-103 to mediate the optimal degradation rate, and thus shows the highest gene transfection efficiency. Furthermore, after treatment, cell viability measurements showed that LC32-103 and all HC32-103 polymers can maintain high levels of cell metabolic activity even at the highest *w*/*w* of 30:1 (>90%), indicating that the polymers are well tolerated by these cells, even 48 h post-treatment. By contrast, PEI/DNA and SuperFect/DNA polyplexes only preserved 60.6% to 70.1% of cell viability compared with untreated cells (Figure S8).

## 4. Conclusions

A series of HC32-103 polymers with varying branching degree were synthesized via the “A2 + B3 + C2” Michael addition reaction. Results show that the branching degree has significant effects on DNA binding, proton buffering capacity, polyplex size, zeta potential, stability, degradation and DNA release rate. Polymer degradation and DNA release rate play key roles in achieving high gene transfection efficiency of HC32-103. The optimal branching degree would lead HC32-103 to exhibit orders of magnitude higher gene transfection efficiency in comparison with the linear counterparts, while maintaining high cell metabolic activity. 

## Supplementary Materials

The following are available online at www.mdpi.com/2073-4360/9/5/161/s1. Table S1: Monomer feed ratios for the synthesis of LC32 and HC32 base polymers, Table S2: GPC results of LC32 and HC32 polymers, Table S3: PDI results of LC32/DNA, HC32/DNA, PEI/DNA and SuperFect/DNA polyplexes with different Polymer/DNA *w*/*w* ratios, Figure S1: Characterization of the LC32-103 and HC32-103 polymers, Figure S2: Cell viability assessment after transfections with polyplexes at different *w*/*w* ratios, Figure S3: Sizes of various polyplexes at different time points after incubation with different concentrations of serum, Figure S4: Sizes of various polyplexes at different time points after incubation with different concentrations of NaCl, Figure S5: Acid-base titration curves of various polymers, Figure S6: Normalized molecular weight of various polymers at different time points measured by GPC after incubation in deionized water, Figure S7: DNA release profiles from various HC32-103/DNA polyplexes, determined by Picogreen assays, Figure S8: Cell viability assessment after transfections with polyplexes at different *w*/*w* ratios: (a) Cell viability of HeLa cells; (b) Cell viability of RDEBK cells.
