# Supplementary material for: Highly Branched Poly(5-amino-1-pentanol-co-1,4-butanediol diacrylate) for High Performance Gene Transfection
Source: Polymers (Basel). 2017 May 1;9(5):161. doi: 10.3390/polym9050161 (PMC6432012; doi:10.3390/polym9050161)
Supplement: Supplementary file 1 [file polymers-09-00161-s001.pdf]

Supplementary Materials: Highly Branched Poly(5-Amino-1-Pentanol-*co*-1,4-Butanediol Diacrylate) for High Performance Gene Transfection

Ming Zeng, Dezhong Zhou, Singwei Ng, Jonathan O'Keeffe Ahern, Fatma Alshehri, Yongsheng Gao, Luca Pierucci, Udo Greiser and Wenxin Wang

Table S1. Monomer feed ratios for the synthesis of LC32 and HC32 base polymers.

Base Polymers	A2 (mmol)	B3 (mmol)	C2 (mmol)	DMSO (g)
LC32	2		2.3	1.1
HC32-10%-ac	2	0.2	2	1.1
HC32-24%-ac	2	0.4	1.7	1.1
HC32-43%-ac	2	0.6	1.4	1.1
HC32-73%-ac	2	0.8	1.1	1.1

Table S2. GPC (Gel permeation chromatography) results of LC32 and HC32 polymers.

Polymers	M_w (kDa)	PDI	MH Alpha
LC32-103	7.6	2.07	0.59
HC32-10%-103	9.6	2.64	0.40
HC32-24%-103	12.8	3.11	0.38
HC32-43%-103	12.0	3.00	0.36
HC32-73%-103	18.3	3.07	0.33

Table S3. PDI results of LC32/DNA, HC32/DNA, PEI/DNA and SuperFect/DNA polyplexes with different Polymer/DNA *w*/*w* ratios.

Polyplexes	PDI		
	10:1	20:1	30:1
LC32-103/DNA	0.23	0.24	0.26
HC32-10%-103/DNA	0.12	0.11	0.08
HC32-24%-103/DNA	0.21	0.18	0.22
HC32-43%-103/DNA	0.23	0.22	0.21
HC32-73%-103/DNA	0.25	0.21	0.24
PEI/DNA	0.43	0.41	0.39
SuperFect/DNA	0.35	0.36	0.34

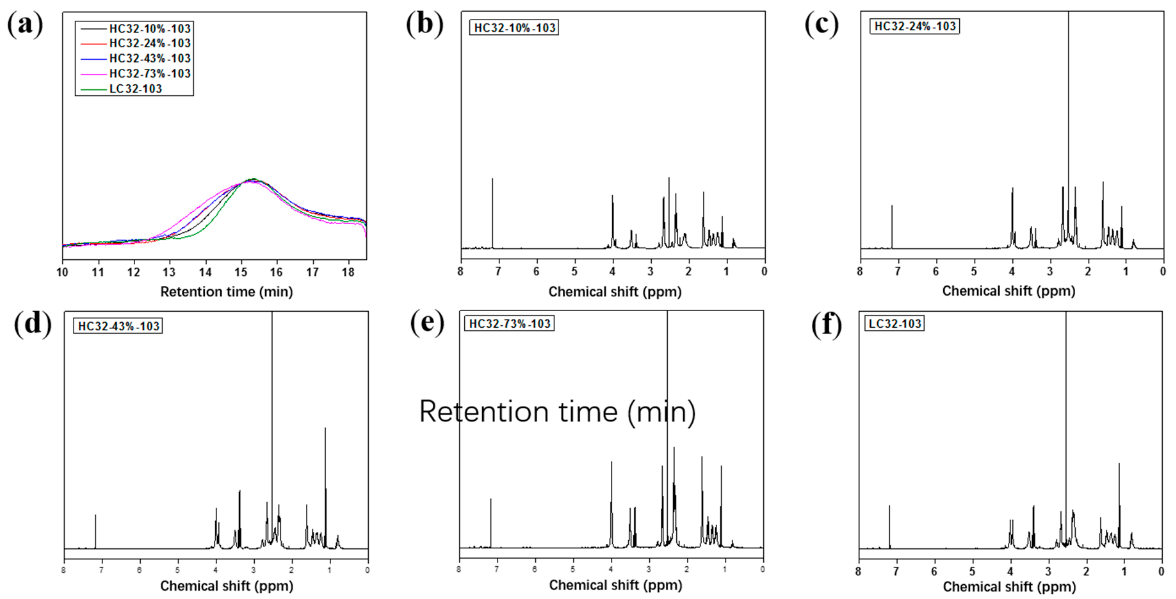

Figure S1. Characterization of LC32-103 and HC32-103 polymers: (**a**) GPC curves of polymers; (**b**–**e**) ^{1}H NMR spectra of HC32-103 polymers with different branched structure; (**f**) ^{1}H NMR spectra of LC32-103 polymer.

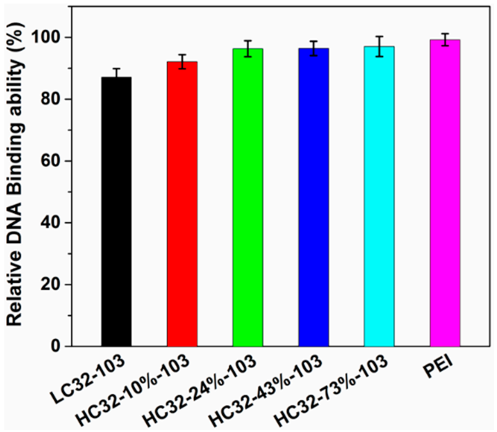

Figure S2. Relative DNA binding affinity of various polymers at the *w*/*w* ratio of 20:1, measured with Picogreen assays.

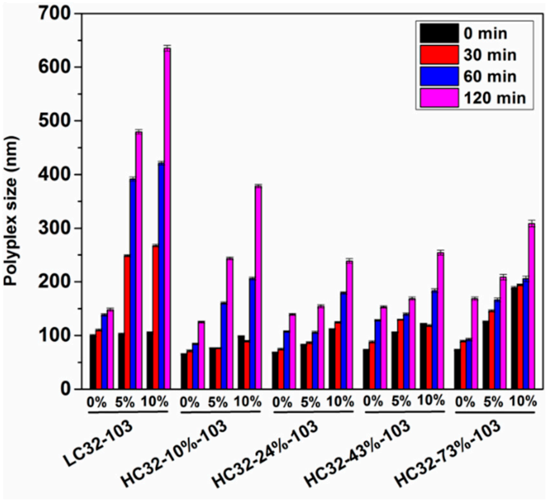

Figure S3. Sizes of various polyplexes at different time points after incubation with different concentrations of serum.

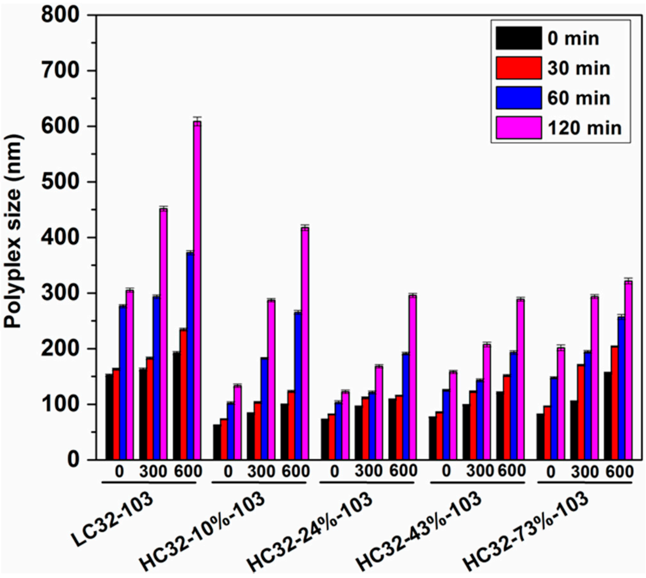

Figure S4. Sizes of various polyplexes at different time points after incubation with different concentrations of NaCl.

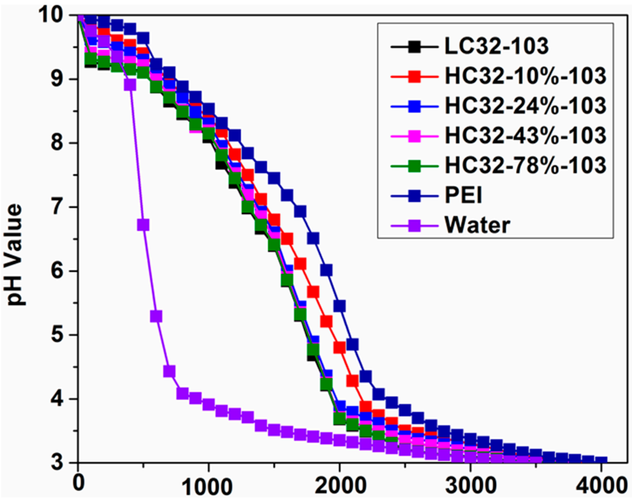

Figure S5. Acid-base titration curves of various polymers.

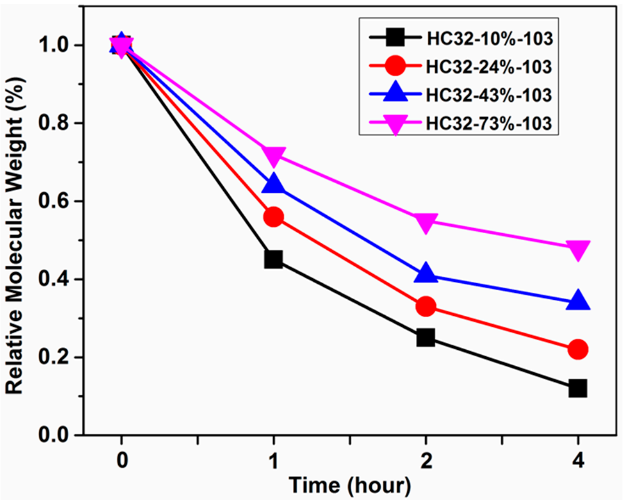

Figure S6. Normalized molecular weight of various polymers at different time points measured by GPC after incubation in deionized water.

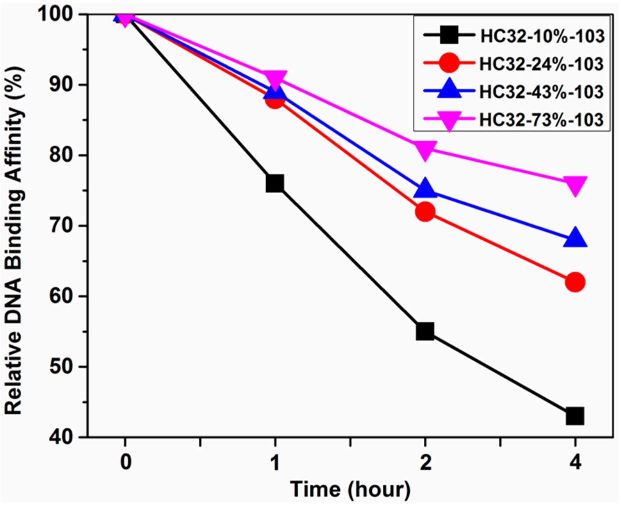

Figure S7. DNA release profiles from various HC32-103/DNA polyplexes, determined by Picogreen assays.

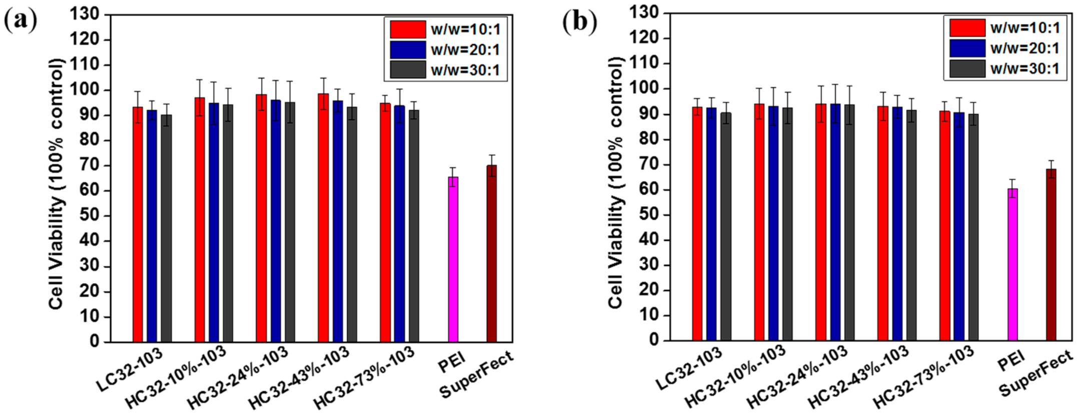

Figure S8. Cell viability assessment after transfections with polyplexes at different *w*/*w* ratios: (**a**) Cell viability of HeLa cells; (**b**) Cell viability of RDEBK cells.

© 2017 by the authors. Licensee MDPI, Basel, Switzerland. This article is an open access article distributed under the terms and conditions of the Creative Commons Attribution (CC BY) license (http://creativecommons.org/licenses/by/4.0/).
